# Numerical analysis of predatory potentiality of *Toxorhynchites splendens* against larval *Aedes albopictus* in laboratory and semi-field conditions

**DOI:** 10.1038/s41598-023-34651-5

**Published:** 2023-05-06

**Authors:** Rajesh Kumar Malla, Koushik Kumar Mandal, Sunanda Burman, Shubhaisi Das, Anupam Ghosh, Goutam Chandra

**Affiliations:** 1 Mosquito Microbiology and Nanotechnology Research Units, Parasitology Laboratory, Department of Zoology, The University of Burdwan, Burdwan, 713104 West Bengal India; 2 Department of Zoology, Banwarilal Bhalotia College, Ushagram, Asansol, 713303 West Bengal India; 3 Department of Zoology, Bankura Christian College, Bankura, 722101 West Bengal India

**Keywords:** Ecology, Zoology

## Abstract

Larvae of the elephant mosquitoes, *Toxorhynchites* spp. (Diptera: Culicidae) are predacious on larvae of other mosquito species and some small aquatic organisms; this predatory behavior can be applied in (mosquito) vector control. The present study examined the feeding behavior of *Toxorhynchites splendens* on *Aedes albopictus* in relation to search area [volume of water (X1)] and prey density (X2), prey instars, predatory preference and larvae's functional response on variable prey densities. Experiments were conducted to determine changes in the feeding activity of *T. splendens* with different search areas and showed that rate of prey consumption was inversely proportional to the search area as evidenced by a negative value of X1 in the regression equation and positively related to prey density. The non-linear polynomial logistic regression estimated a significant linear parameter (P_1_ < 0) for the functional response analysis suggesting a Type II functional response. Differences in feeding response related to the different combinations of prey instars were statistically not significant (p > 0.05), expressing that all the instars of prey were equally susceptible to the predator. *Toxorhynchites splendens* preferred to consume *Ae. albopictus* larvae rather than *Tubifex* when supplied together as a food source.

## Introduction

Most of the world's tropical and sub-tropical countries face high mortality and morbidity associated with various life-threatening vector-borne diseases like malaria, filariasis, and other viral infections^1^. Different species of mosquitoes that belong to the genus *Aedes* act as potent vectors for transmitting viruses like dengue, chikungunya, yellow fever, zika, etc.^2^. Disease burden is now increasing in endemic areas due to an almost geometric increase in the human population density, rapid urbanization, absence of optimal surveillance programs, and the appearance of resistance in the mosquito population to the commonly used chemical insecticides, limited application of proper vector control measures and general ignorance of the common people to adopt appropriate prophylactic measures^3^.

Vector control is the most effective measure for combating harmful vector-borne diseases^4^. Vector control approaches include fumigation, spraying of chemical insecticides or application of bacterial formulations, using permethrin-treated bed nets, and introducing larvivorous fishes such as Guppy and *Gambusia* in the temporary or permanent aquatic mosquito breeding habitats^5,6^. However, nowadays, alternative vector control strategies using biological agents other than fish that feed on the immature stages of mosquitoes (both larva and pupa) have gained interest due to the eco-friendly nature of these applications^7^.

Larvae of the mosquito genus *Toxorhynchites* (Diptera: Culicidae) are predacious on larvae of other mosquito species and other small aquatic organisms^8^. Paine^9^ first suggested *Toxorhynchites* species as a biocontrol agent for mosquitoes in the Pacific Islands. *Toxorhynchites* have been reported as an effective biological control agent for container-breeder *Aedes aegypti* as well as *Aedes albopictus*^10^*.* Predatory efficiency of different species of *Toxorhynchites* larvae against *Culex quinquefasciatus* and *Armigeres subalbatus* mosquito larvae has been reported^11^.

 Focks et al.^12^ reported on the carnivorous habit of all larval instars of *Toxorhynchites* spp., which could consume the immature life stages (larva and pupa) of other mosquito species. Rubio and Ayesta^13^ established that this species could exist in a very long larval life lasting for three weeks or more, their eggs could withstand desiccation, and larvae could withstand starvation like *Aedes* larvae. Corbet and Griffiths^14^ and Taylor^15^ showed a very peculiar habit of *Toxorhynchites*, where the larvae nearing pupation exhibited a "killing without eating" behavior in which they used to kill a large number of prey mosquito larvae present in the common habitat. They naturally breed in the same habitat as that of *Aedes* sp., i.e., earthen pots, unused containers, small water tanks etc.^16^.

However, before recommending the mass releases of this predatory mosquito through inoculation or augmentation in the temporary or permanent water bodies where immatures of vector mosquitoes exist, a detailed numerical analysis (in terms of functional response) of the predator–prey relationship is important. The objective of the present study was to observe the feeding behavior of *Toxorhynchites splendens* in relation to the search area (volume of water) and to investigate the effect of variable prey (*Ae. albopictus*) density, different prey instars, and alternative food on the functional response of *T. splendens* larvae. Larval feeding pattern in semi-field condition was also examined.

## Materials and methods

### Collection of larvae of prey and predator species

Larvae of *Ae. albopictus* were collected from discarded water-filled tires alongside Grand Trunk road passing through Burdwan town, Purba Bardhaman, West Bengal (23°13′57.0468′′ N and 87°51′48.3084′′ E) during early November 2018. Then collected larvae were transferred to plastic trays, filled with normal tap water (aprox.100 larvae per tray) and maintained in the laboratory at 27 ± 2 °C and 60–70 percent relative humidity with an artificial diet containing powder of Brewer yeast, dog biscuits, and algae mixture at 3:1:1 ratio^17^. Commercially available algal powder (*Spirulina* sp.) was used as a high protein source in the food mixture. We used the required amount of food (0.5 mg/larvae) and poured it into the larval tray after thoroughly dissolving it in 5–10 ml of water. The water remained translucent after the food was added. For 1st and 2nd instar larvae, the foods were offered once a day, twice for 3rd and thrice for 4th instar larvae. Species *Aedes albopictus* was affiliated following the key specified by Rueda^18^. Acclimatized (for 4–5 days) prey larvae were carefully taken from the rearing trays and used for different bioassay experiments.

Twenty earthen pots, each of which half filled with one liter pond water, were placed in some bushy areas on the campus of The University of Burdwan, West Bengal, India. Wild *T. splendens* mosquitoes laid eggs in the earthen pots, and after 2–3 days, larvae hatched out from the laid eggs. Larvae were collected carefully, brought to the laboratory, and reared in laboratory conditions. Larvae were placed in plastic trays at 20 ℃ ± 2 °C temperature, and *Tubifex* was provided as food. *T. splendens* larvae were kept separately in containers on attaining 2nd instar to avoid cannibalism among them. After 15–20 days, adults emerged. Identification of adult *Toxorhynchites splendens* was made according to Rattanarithikul et al.^19^. Fresh batches of next-generation laboratory-bred larvae were used for further experiments.

### Collection of *Tubifex*

*Tubifex*, which was used as alternative food in one experiment, was collected alive from the aquarium seller of Burdwan town. It is generally used as food for ornamental fishes.

### Different experimental setups

At the outset, a temperature-dependent study on the life cycle stages and feeding behavior of 3rd instar *T. splendens* larvae (predator) was conducted on 3rd instar *Ae. albopictus* larvae (prey) at 27° ± 1 °C and 20° ± 1 °C under laboratory conditions. This preliminary study was carried out to determine if the temperature has any effect on the duration of the life stages of the predator. In each case, 100 prey larvae were provided to one predator larva in 100 ml of water in a 250 ml glass beaker and repeated for three consecutive days. Average span/ duration of 3rd instar larva of *T. splendens* (development time from 3rd instar to 4th instar) as well as average daily feeding rate of *T. splendens* on *Ae. albopictus* was determined at both temperature setups. Interestingly, the span of the 3rd instar of *T. splendens* larvae was longer at 20 °C ± 1 °C temperature compared to that of at 27° ± 1 °C, but the average daily feeding rate was more or less the same. To fulfill the objectives of the second experiment, which continued for nine consecutive days, all further laboratory experiments were conducted at 20 °C ± 1 °C.

In the first experiment, changes in feeding activity with different search areas (volume of water) (X1) and with changing prey density (the number of prey given) (X2) were determined. Six 250 ml glass containers were taken. The first three containers were filled with 50 ml, 100 ml, and 200 ml of regular tap water. Fifty 3rd instar *Ae. albopictus* larvae along with a 3rd instar *T. splendens* larva, were introduced in each container. The other three containers were also filled with 50 ml, 100 ml, and 200 ml of tap water, but this time 100 prey larvae of 3rd instar and a 3rd instar predator larvae were kept in each container. After 24 h, the number of larvae consumed by the predator was counted. Experiments were done thrice on three separate days with different prey and predator larvae of the same instar.

In the second experiment, the feeding rate of a single 3rd instar larva of *T. splendens* was evaluated against the increasing prey densities in a glass beaker having 100 ml of water. In the beaker, a single predatory larva of *T. splendens* was used as predator, and increasing numbers of prey items (20, 30, 40, 50, 60, 70, 80, 90, and 100 3rd instar larvae of *Ae. albopictus*/day) were provided to it for predation. The experiment was conducted for nine consecutive days for the above mentioned nine prey densities. After each day, the feeding rate was recorded for each larval density, and fresh prey larvae were added with the remaining number of prey in the beaker to maintain the next prey density. The experiment with each larval density was repeated thrice.

The third experiment was designed to determine the effect of prey instars on the functional response of *T. splendens* larvae. For this experiment, six 250 ml glass containers were taken, and each container was filled with 100 ml of tap water. Then prey larvae of two different instars (25 of each instar) together were introduced in each (a total of 50 larvae). All possible combinations like 1st and 2nd, 1st and 3rd, 1st and 4th, 2nd and 3rd, 3rd and 4th, and 2nd and 4th of prey instars were given in containers 1–6, respectively. One 3rd instar *T. splendens* was placed in each container. Numbers of prey (instar-wise) consumed by the predator were observed and recorded after 24 h. The experiments were repeated three times on three different days with different sets of prey and predator of almost similar size.

In the fourth experiment, to determine feeding response in the presence of alternative food, 25 *Ae. albopictus* larvae and 25 *Tubifex* sp. were introduced in a 250 ml glass container containing 100 ml of tap water. *Tubifex* sp. was obtained from the aquarium culture of a seller in living conditions. A 3rd instar *T. splendens* larva was put into the glass container as a predator. The number of *Aedes* larvae and *Tubifex* sp. that the predator consumed were recorded after 24 h. We have selected *Tubifex* as alternative food because it is a natural food item of *Toxorhynchites* larvae^20^. The experiment was repeated three times on three different days with different sets of prey and predator.

A semi-field experiment was carried out as the fifth experiment in the garden of the Burdwan University campus for six days (27th July, 2019–1st August, 2019). To obtain sufficient predator and prey larvae for this experiment, ten earthen pots of one-liter capacity were placed in an open area on the campus and allowed to fill with rainwater in the rainy season and kept under observation for laying eggs by *T. splendens* and *Ae. albopictus*. Both *T. splendens* and *Ae. albopictus* were found to have container breeding habits. Within 10 days, we got the required number of singly laid eggs and 1st instar larvae of *Toxorhynchites* and *Aedes*. A few egg rafts laid by any species of *Culex* were discarded. *Aedes* larvae were identified as *Aedes albopictus* following the key of Rueda^18^, and accordingly, *Toxorhynchites* larvae were identified as *Toxorhynchites splendens*. A late 3rd instar *T. splendens* larva along with 250 *Ae. albopictus* larvae (2nd instars, collected from earthen pots) were released in the experimental pot on the 1st day (27th July). The number of prey larvae consumed was counted after every 24 h. Consumed prey larvae were compensated each day to maintain constant prey density after counting prey numbers. The experiment was triplicated in three separate pots. Obtained data were applied to the given equation to determine the clearance rate (CR) following Gilbert and Burns^21^ with some modifications:

$$CR=\frac{\mathrm{V}(\mathrm{lnP})}{\mathrm{TN}}$$

where CR, clearance rate of predator (number of prey eaten or killed /liter/day/predator); V, volume of water (L); p, number of prey consumed or killed; T, time in days and N, Number of predators.

### Statistical analysis

For the statistical analysis of the first experiment, i.e., to observe the changes in feeding rate (Y) on search area (X1) and prey densities (X2), a multiple regression equation was computed.

The functional response of *T. splendens* was analyzed against different densities of mosquito larvae. The type of response was established by non-linear polynomial logistic regression equations of the proportion of prey eaten function of initial prey density (*N*_*a*_* /N*_*0*_) as described by the random attack equation of Juliano^22^:

$$\frac{{N}_{a}}{{N}_{0}}= \frac{\mathrm{exp}\left({P}_{0}+{P}_{1}{N}_{0}+{P}_{2}{N}_{0}^{2}+{P}_{3}{N}_{0}^{3}\right)}{1+exp\left({P}_{0}+{P}_{1}{N}_{0}+{P}_{2}{N}_{0}^{2}+{P}_{3}{N}_{0}^{3}\right)}$$

where *N*_*a*_, the number of prey eaten; *N*_*0*_, the initial prey number provided. P_0,_ P_1_, P_2,_ and P_3_ are the intercept, linear, quadratic, and cubic coefficients, respectively. Maximum likelihood estimates of parameters P_0_–P_3_ were calculated by logistic regression to a binomial variable that equaled 0 for surviving preys and 1 for consumed preys. As the functional response represents Type-II, the associated parameters, i.e., attack rate and handling time, were calculated using Holling's Disc equation (1959)^23^ as follows:

$${N}_{a}=\frac{a{N}_{0}T}{1+a{N}_{0}{T}_{h}}$$

where *a*, the attack rate constant; *T*, total time available (here, 24 h), and *T*_*h*_, handling time per prey. The attack rate estimates the rate of prey consumption as a function of variable prey densities and handling time calculated by the time required to attack and consume prey. In the equation, *N*_*0*_ is the independent variable, and *Na/N*_*0*_ is the outcome variable. "MS Excel 2007" and "R" (Version 4.2.2) statistical software were used for statistical analysis. For the calculation of attack rate and the handling time, at first, we have linearized the Holling's Disc equation as 1/Ha = (1/a)/(1/HT) + *T*_*h*_/T, which is equivalent to the straight-line equation; y = α + βx. Now, the handling time (*T*_*h*_) can be determined by plotting the data of H/Ha versus H and multiplying the total exposition time (T) by the angular coefficient of this straight line (β). The attack rate (a) corresponds to the intercept of the straight line (1/α).

For the prey instars preference analysis paired t-test was performed for each combination.

## Results

From the temperature-dependent study on the life cycle stages and feeding behavior of 3rd instar *T. splendens* larvae (predator), we have found that the average duration of the 3rd instar of *T. splendens* larvae was longer (10–11 days) at 20 °C ± 1 °C temperature than that of (7–8 days) at 27° ± 1 °C, but the average daily feeding rate was more or less same.

The first experiment, which was to determine changes in feeding activity with different search areas (volume of water) (X1) and with changing prey density (number of prey given) (X2), showed many outcomes that are presented in Table 1. Regression equation analysis revealed that the feeding rate (Y) was inversely proportional to the search area (X1), as evidenced by the negative value and positively related to the prey densities (X2). The respective R^2^ values were close to 1(R^2^ = 0.957).

**Table 1:** Number of *Aedes albopictus* consumed by *Toxorhynchites splendens* in different volumes of search area (volume of water) and three prey densities (number of prey).

Number of predators	Volume of water (ml) (X1)	Number of prey given (X2)	Average number of prey consumed (Y) ± standard error	Regression equation
1	50	50	30.33 ± 2.51	Y = − 0.08689X1 + 0.0488X2 + 31.92 (R^2^ = 0.957)
	100	50	25.67 ± 2.51	
	200	50	16.67 ± 2.08	
	200	100	20.33 ± 2.08	
	100	100	26.33 ± 1.52	
	50	100	33.33 ± 2.08	

**Figure 1:**
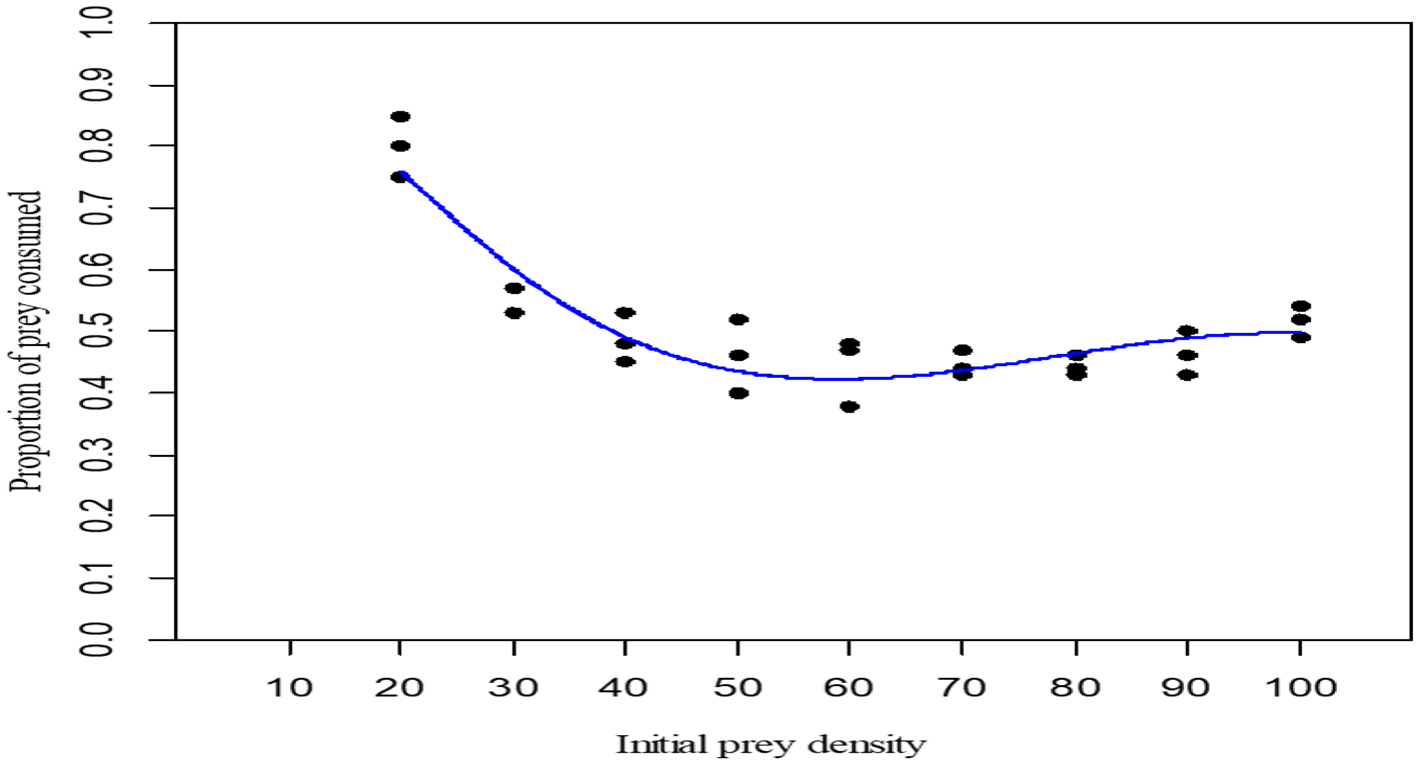
Proportion of prey (*Aedes albopictus* larvae) consumed with increasing prey densities.

The result of the second experiment, which was to assess the functional response changes in relation to increasing prey densities, is shown in Table 2. Different functional response parameters and coefficients are presented in Table 3. Since P_1_ < 0, the proportion of prey consumed declined monotonically with the initial number of prey given, and thus it showed a type II functional response (Fig. 1). Accordingly, the Holling Disc equation was used for the estimation of instantaneous attack rate (A) and handling time (*T*_*h*_). Here the attack rate was 0.803 per hour, and the handling time was 6.38 min. The respective R^2^ values were close to 1(R^2^ = 0.936), predicting a good fit of Holling Disc rather than the Random predator equation.

**Table2:** Mean number of *Aedes albopictus* larvae consumed by predator (*Toxorhynchites splendens* larvae) with increasing prey densities.

Initial Prey density	Number of Larvae consumed			The mean number of larvae eaten	Standard deviation ( ±)	Standard error ( ±)	Equation of type II response
	Day 1	Day 2	Day 3				
20	17	16	15	16.00	1.00	0.58	y = 0.000x^2^−0.022x + 1.158 R^2^ = 0.936
30	17	16	16	16.33	0.58	0.33	
40	18	21	19	19.33	1.53	0.88	
50	20	26	23	23.00	3.00	1.73	
60	23	29	28	26.67	3.21	1.86	
70	30	33	31	31.33	1.53	0.88	
80	34	37	35	35.33	1.53	0.88	
90	41	45	39	41.67	3.06	1.76	
100	52	54	49	51.67	2.52	1.45

**Table 3:** Estimated values of functional response parameters; here, P_0,_ P_1_, P_2,_ and P_3_ are the intercept, linear, quadratic, and cubic coefficients, respectively.

Search area (ml)	Parameters	Estimate	Std. error (S.E.)	t-value	Pr( >|t|)
100	P_0_	3.729e+00	5.518e−01	6.758	6.820e−07
	P_1_	− 1.717e−01	3.237e−02	− 5.304	2.200e−05
	P_2_	2.332e−03	5.712e−04	4.083	0.000458
	P_3_	− 9.891e−06	3.093e−06	− 3.198	0.003995

**Figure 2:**
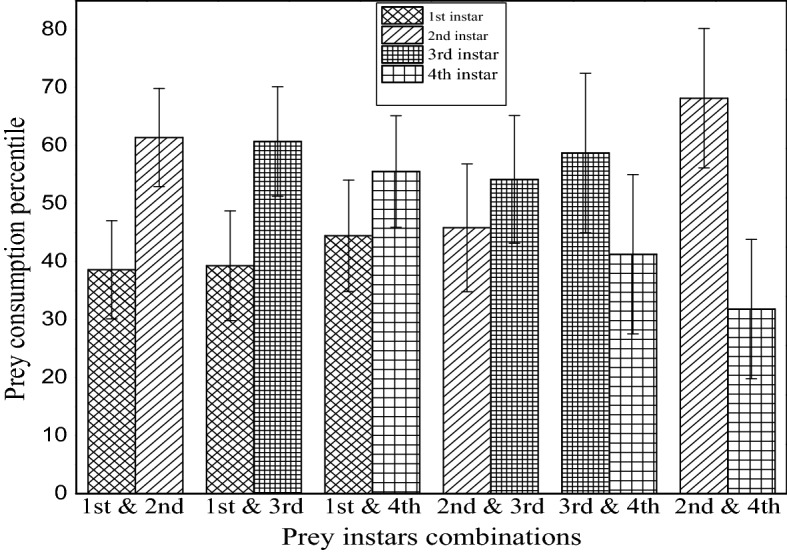
Consumption rate of prey species when given in different combinations of instars of prey.

Figure 2 displays the result of changes in feeding habits with different prey instars of all six combinations. It showed the feeding percentage of their diet on different instar of prey larvae in different instar combinations. The highest consumption rate (68.17%) of predator larvae in their diet was detected against 2nd instar prey larvae when the prey instar combination of 2nd and 4th instar larvae of prey was used. From Fig. 2, maximum consumption was noted apparently against 2nd and 3rd instar larvae in different combinations. Moreover, the prey preferences fluctuated in every combination examined. As in all six combinations, the computed p-values were higher than the significance level alpha (α) = 0.05. The null hypothesis (H0) was accepted, indicating no significant difference in feeding rate in relation to prey instars.

**Figure 3:**
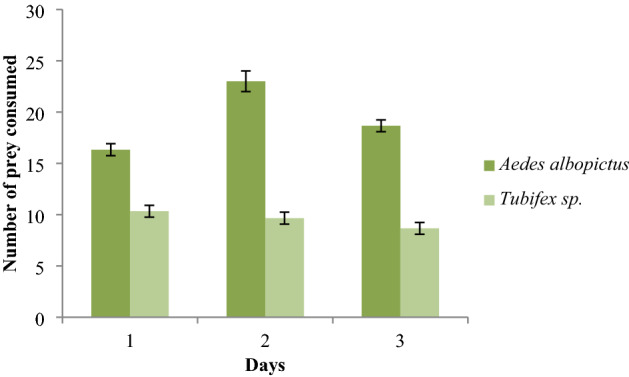
Average number of *Aedes albopictus* larvae and *Tubifex* consumed by *Toxorhynchites splendens.*

The feeding activity of *T. splendens* in the presence of alternative food (*Tubifex* sp.) is shown in Fig. 3. It is observed that *T. splendens* preferred to consume more *Ae. albopictus* larvae than *Tubifex* in their diet when both were supplied as a food source. Thus, a clear prey preference of *T. splendens* on *Ae. albopictus* was established in this experiment.

**Figure 4:**
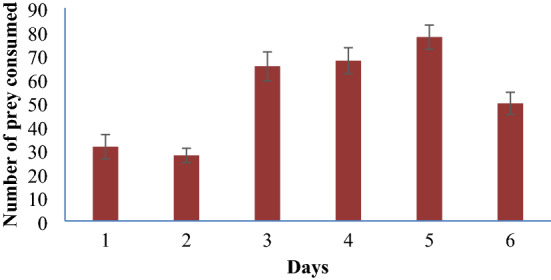
Number of larvae consumed by *Toxorhynchites splendens* larvae in semi-field condition.

In all the earthen pots set as ovitrap for the semi-field experiment, both *T*. *splendens* and *Ae. albopictus* egg and larvae were found. In semi-field conditions, the late 3rd instar *T. splendens* larva showed a significant feeding performance, as presented in Fig. 4. The clearance rates in semi-field conditions are presented in Table 4. The value ranged from 3.29 to 4.34 prey larvae/liter/day/predator. Initially (on days 1 and 2), the clearance rate is lower, and the highest value is observed on day 5, following the lower clearance rate on day 6.

**Table 4:** Clearance rate (CR) values on successive days during the semi-field experiment.

Day of exposure	CR value
1st	3.42
2nd	3.29
3rd	4.17
4th	4.20
5th	4.34
6th	3.98

## Discussion

*Toxorhynchites* sp*.* (Diptera: Culicidae) is the only genus within the tribe Toxorhynchitini and consists of about 90 species^24^. *Toxorhynchites* are commonly known as 'elephant mosquitoes' due to their larger size than the adult of other species of mosquitoes and trunk like proboscis adapted for nectar feeding^20^. *Toxorhynchites* are mainly distributed in the forest areas of tropical countries and some temperate areas of the world between about 35° north and 35° south^25^. The adults are strictly nectarivorous and deposit their eggs primarily in sylvatic habitats, like water-filled tree holes, bamboo stumps, and coconut husks, as well as in a variety of artificial containers, sewage drains, discarded cans, tires, and ricefield^11,20^. The larval instars are carnivorous in nature, preying on many smaller aquatic invertebrates, and even show cannibalistic behavior in the absence of preferred food items^24^. Crustaceans, nymphs of dragonflies and damselflies, aquatic bugs like backswimmers, giant water bugs, water boatmen, larvivorous fishes, tadpoles, etc. have been reported as mosquito larval predators in general^26^. Though *Toxorhynchites* itself is a mosquito genus, it is a general assumption that above mentioned predators may feed upon *Toxorhynchite*s species if they share common habitat. But no report on predation of general larval feeders specifically on *Toxorhynchites* is not available. *Ae. albopictus*, a major rural vector responsible for the transmission of dengue, zika, west nile fevers, chikungunya, etc., and found in the tropical countries of the world. The adults breed in different types of rainwater-filled natural and artificial containers, tires and tubes, and micro water bodies like tree holes, bamboo holes, earthen pots, etc^27^. The similarity in the selection of habitats for oviposition of *Toxorhynchites* and *Ae. albopictus* is advantageous for the effective management of the latter one by the application of *Toxorhynchites* as an effective biocontrol tool^26^.

All the organisms in nature are interconnected by each other directly or indirectly through different ecological aspects. A community gets stability through various interactions among the organisms. Functional response analysis is a kind of analysis of interaction among predator and prey species. It describes changes in the consumption rate of predators in response to the changes in prey density^28^. Mondal et al.^29^ previously described the effects of temperature and search area on the functional response of *Anisops sardea* against larvae of *Anopheles stephensi*. The present experiments help to understand the interaction between *T. splendens* mosquito larvae as a predator and *Ae. albopictus* larvae as prey, and understanding this prey-predator relationship is necessary before applying *T. splendens* as a mosquito bio-control agent in the field. *T. splendens* could not perform satisfactorily for the control of *An. polynesiensis*, since their oviposition sites did not coincide^30^. However, *T. splendens* and *Ae. albopictus* breed in the same habitats, where *T. splendens* naturally predate on *Aedes* larvae in a target-specific manner^31^.

According to Yasuda^32^, the feeding activity of the predatory mosquito, *Toxorhynchites towadensis* elevated with increasing prey density. It is known that most of the single predator-single prey functional response curve using insect predator generally exhibits Type II response^33,34^. The result obtained in the current study implies that *T. splendens* larvae exhibit type II response against the *Ae. albopictus* larva in different prey densities. Here it is observed that the larval consumption rate decreases with the increasing volume of the search area.

Conversely, the feeding rate of *Toxorhynchites splendens* larvae enhances with increasing prey density. Therefore, a change in feeding rate in the different search areas is an essential parameter for consideration during field application. Thus, this study implies that during field application, *T. splendens* larvae should be released by considering the actual volume of the target area and prey density in that area.

According to Steffan and Evenhuis^24^ and Tikasingh and Martinez^35^ the feeding rate and total prey consumption during larval development of *Toxorhynchites* sp. depend on several factors, including prey size. In the present study, instar preference analysis states that although there are small differences observed in all six combinations of prey larvae, the differences are not statistically significant (p > 0.05), which also reveals that larva of *T. splendens* prefer all the prey instars more or less equally. So, as a predator, *T. splendens* larva is equally effective against all four prey instars.

In the presence of *Tubifex* sp. as alternative food, *T. splendens* larva consumed more *Ae. albopictus* larva, indicating the predatory preference of *T. splendens* toward the larvae of *Ae. albopictus* (Fig. 3). It is generally believed that mosquito larvae are the main prey of *Toxorhynchites* sp., but a study done by Campos and Lounibos^36^ showed that the *T. rutilus* prefer ostracods and chironomid larvae and only 5–6% of their preys are mosquito larvae.

As eggs and larvae of both *T. splendens* and *Ae. albopictus* were found in sufficient number in all the ovitraps, no oviposition avoidance and temporal segregation between these two species are apparent. In semi-field conditions, the larval consumption rate is also noticeable. The CR values in all six days express the ability of *T. splendens* larva to be established as an excellent bio-enemy of vector mosquito species, *Ae. albopictus*. The high clearance rate and low handling time are general characteristics of a successful biocontrol agent in field conditions^37^. The clearance rates in the initial two days are minimum, probably for acclimatization of predators in the semi-field habitat, and after that, it moves upward and reaches its maximum value on day five.

The average daily consumption rate in semi-field conditions is 52.66 against 2nd instar *Ae albopictus* mosquito larvae. So, the yearly estimated consumption rate of a single 3rd instar *T. splendens* larva over 2nd instar *Ae. albopictus* larva is approximately 19,220. The experiments are done with a realistic prey density (*Ae. albopictus*), i.e., 200–300 larvae per liter of water in the natural habitats, which indicate that the feeding rate of *T. splendens* larvae even in field condition without interventions of man will be more or less same as the semi-field condition. Thus, using *T. splendens* larvae as a natural predator can be an effective biological tool for controlling the population of dengue vector *Ae. albopictus* in endemic areas.

## Conclusion

Thus, the present study concludes that the release of *T. splendens* mosquito larvae in nature will be a fruitful measure in eco-friendly, target-specific, and effective dengue control. The present study also describes how search area, prey density, instars differences, and presence of alternative food influence the consumption rate of *T. splendens* in detail. This study will conceivably facilitate the biological control program of vector mosquitoes that breed in the same habitats as *Toxorhynchites*. Biological control of *Ae.albopictus* is feasible with its natural predator *T. splendens,* in endemic regions.

## Data Availability

All data generated or analyzed during this study are included in this published article.
